# Comparative ovicidal activity of *Moringa oleifera* leaf extracts on *Fasciola gigantica* eggs

**DOI:** 10.14202/vetworld.2018.215-220

**Published:** 2018-02-19

**Authors:** Ahmed G. Hegazi, Kadria N. Abdel Megeed, Soad E. Hassan, M. M. Abdelaziz, Nagwa I. Toaleb, Eman E. El Shanawany, Dina Aboelsoued

**Affiliations:** 1 Department of Zoonotic Diseases, National Research Centre, Dokki, Giza, Egypt; 2 Department of Parasitology and Animal Diseases, National Research Centre, Dokki, Giza, Egypt

**Keywords:** *Fasciola gigantica*, leaf extract, *Moringa oleifera*, nitroxynil, ovicidal activity

## Abstract

**Background:**

Fasciolosis is an important zoonotic disease affecting the productive performance of farm animals in Egypt.

**Aim:**

The aim of the present study was comparing the ovicidal effect of different extracts as an alcoholic (Methanolic and Ethanolic) and aqueous *Moringa oleifera* leaf extracts on *Fasciola gigantica* non-embryonated and developed eggs.

**Materials and Methods:**

Tested concentrations of extracts ranged from 12.5 to 800 mg/ml. Nitroxynil was used as reference drug with a dose of 100 mg/ml.

**Results:**

*M. oleifera* alcoholic and aqueous extracts showed a concentration-dependent ovicidal effect on *F. gigantica* non-embryonated and developed eggs. Based on LC_50_ values, water extract showed the highest ovicidal activity since it registered the lowest values of 2.6 mg/ml on non-embryonated eggs. Non-embryonated eggs were more susceptible to aqueous extract than developed eggs. On the other hand, the developed eggs were more susceptible to ethanolic extract than non-embryonated eggs even the lowest LC_50_ (12.38 mg/ml).

**Conclusion:**

*M. oleifera* leaf extracts especially aqueous extract could be a promising step in the field of controlling fascioliasis. Further, *in vivo* studies are needed to enlighten the therapeutic potential of *M. oleifera* extracts in treating *F. gigantica* infection.

## Introduction

Fasciolosis is considered one of the most important helminthic diseases affecting livestock in many countries around the world. It is commonly found in sheep and cattle [1]. Bovine fasciolosis usually had no visible clinical signs. However, if the cattle infection was chronic, it might cause weight gain loss, reduction in the milk yield, and fertility problems [2,3].

Many available chemotherapeutic anthelmintics had side effects on the host, and it is necessary to decrease the use of these drugs for parasitic control, not only for their resistance but also because of growing concerns about the adverse consequences on the ecosystem. Naturally produced plant anthelmintics offer an alternative that overcomes some of these effects and is both sustainable and environmentally acceptable [4]. Around the world, new plants having medicinal properties against parasites of ruminants had been explored, and they had shown good results [5]. *Moringa oleifera* is considered as one of the most useful trees for the reason that every part has many beneficial properties [6]. *M. oleifera* is commonly known as Drumstick and contains many active compounds such as alkaloids, flavonoids, saponins, tannins, and triterpenoids [7]. Extracts from this plant have several pharmacological effects such as anthelminthic [8-10], anti-inflammatory, antimicrobial, and antioxidant [11].

The aim of the current study was to compare the ovicidal effect of different extracts as an alcoholic (methanol and ethanol) and aqueous *M. oleifera* leaf extracts on *Fasciola gigantica* non-embryonated and developed eggs.

## Materials and Methods

### Ethical approval

*F. gigantica* eggs were recovered from gallbladders of cattle and buffaloes slaughtered in the government abattoir according to governmental regulations.

### Collection of plant materials

*Moringa* leaves were collected from Pilbis, Sharqia Governorate, Egypt, kindly by Prof. Aboelfetoh M Abdalla, Moringa Unit, National Research Centre. The leaves were cleaned with tap water 3 times then by phosphate buffer (pH.7.4).

### Preparation of extracts

#### Alcoholic extract

150 g of *M. oleifera* green leaves were separately extracted in 1500 ml of 80% ethanol (ethanolic extract) and 80% methanol (methanolic extract), then manually shaken for 30 min and allowed to stand with continuous shaking at a shaking water bath for 3 days. After that, solutions were filtered with sterile filter papers (Whatman No.1) into a clean conical flask and dried under reduced pressure in a rotary evaporator at a temperature below 50°C and stored at 4°C until use.

#### Aqueous extract

10 g of *M. oleifera* shade dried leaves were ground into powder and extracted by maceration in 1000 ml of double distilled water accompanied by stirring for 24 h in cold conditions 4°C then filtered with sterile filter papers (Whatman No.1) into a clean conical flask and stored at −20°C until use.

### Drug of choice

Nitroxynil 25% (Devomor^®^) (Arabcomed for El-Nehesi Company, Egypt) and the dose was 100 mg/ml.

### Collection of F. gigantica eggs

*F. gigantica* eggs were recovered from bile of the gallbladders of infected cattle and buffaloes slaughtered in Cairo local abattoir, Egypt. Gallbladders were washed and examined individually. Each gallbladder was evacuated separately in the 1 L cylinder, mixed with tap water, left to sediment and then the supernatant was decanted without disturbance of the sediment. This process was repeated 3-5 times. The clean *F. gigantica* eggs were collected after sedimentation and stored in distilled water to be used as fresh as possible.

### Ovicidal effect of M. oleifera on F. gigantica eggs

Two experiments were involved in the present study; one was for non-embryonated and the other was for developed (morula stage) *F. gigantica* eggs. The alcoholic extracts concentrations were 12.5, 25, 50, 100, and 200 mg/ml, while in aqueous extract they were 100, 200, 400, and 800 mg/ml. Three replicates of every dilution were prepared in each treatment. Each replicate was contained 10 ml double distilled water and about 100 *F. gigantica* eggs and the selected extract concentration. Three replicates of 100 mg/ml nitroxynil drug were tested. Another group of eggs were incubated in distilled water and served as control group. The Petri dishes were incubated at 28°C in dark environment for 14 days. The numbers of hatched and non-developed eggs were counted*. F. gigantica* eggs and their developmental stages including the morula, eyespot stage or embryonated eggs and hatched eggs were identified as previously described [12].

The percentage of hatched and developed eggs for each treatment was calculated [13], and hatching ratio was calculated according to Canevari *et al*. [14].

### Statistical analysis

Data of ovicidal effect of different treatment groups were analyzed for the means and standard deviations. The significance of the results was evaluated using independent sample t-test and analysis of variance and Duncan test using Statistical Package for the Social Sciences computer programs [15].

## Results

**Figure-1:**
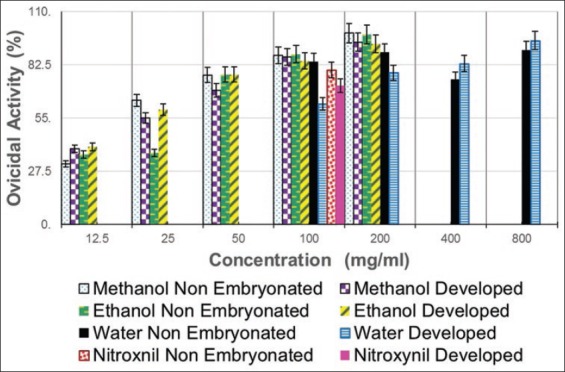
Ovicidal activities of *Moringa* extracts and nitroxynil on non-embryonated and developed *Fasciola* eggs.

**Figure-2:**
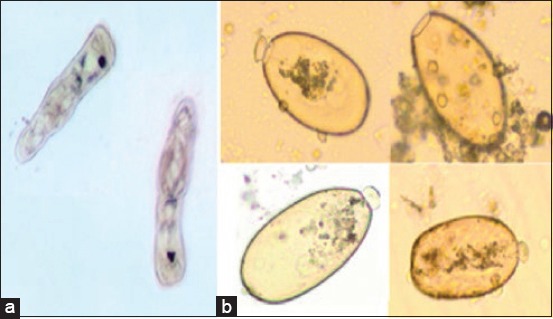
Control untreated *Fasciola gigantica* eggs. (a): Hatched miracidia with eye spots, (b): Hatched eggs (empty eggs with opened operculum) (40×).

**Figure-3:**
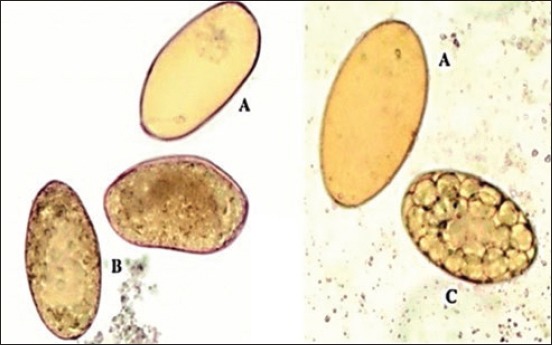
*Moringa* treated *Fasciola* eggs (methanol extract) at highest concentration 200 mg/ml: (A) Empty *Fasciola* eggs with a closed operculum, (B) dead eggs with lysed embryo, (C) eggs of early development (40×).

**Figure-4:**
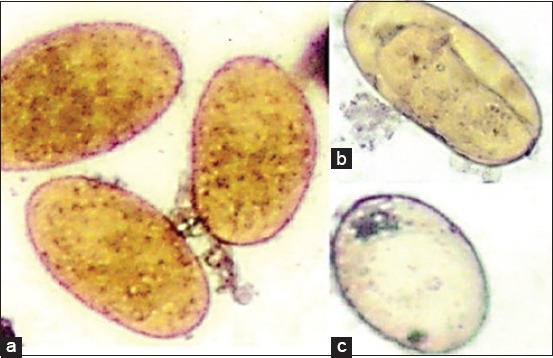
*Moringa* treated *Fasciola* eggs (Ethanol extract) at highest concentration 200 mg/ml: (a) Degenerated eggs, (b) dead miracidium inside the egg, (c) empty *Fasciola* egg with a closed operculum (40×).

**Figure-5:**
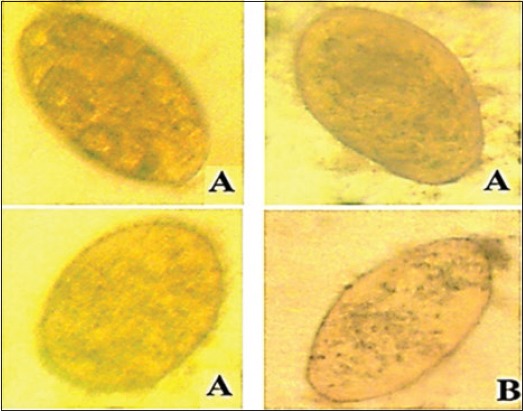
*Moringa* treated *Fasciola* eggs (water extract) at highest concentration 800 mg/ml: (A) Degenerated eggs and (B) empty *Fasciola* egg with a closed operculum (40×).

**Figure-6:**
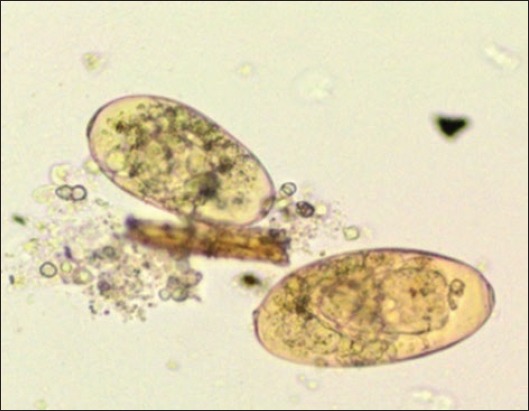
Nitroxynil treated *Fasciola* eggs at concentration 100 mg/ml: Degenerated eggs (40×).

The ovicidal effects of the different *M. oleifera* extract on the *F. gigantica* eggs are displayed in Tables-1 and 2. *M. oleifera* alcoholic and aqueous extracts, as well as nitroxynil, showed an ovicidal effect on *Fasciola* non-embryonated and developed eggs (Figures 1-6). The effect of these extracts on eggs was concentration dependent. There were no statistically significant differences between the ovicidal activity of *M. oleifera* methanolic and ethanolic extracts in non-embryonated eggs. Water extract at a concentration of 800 mg/ml had the same effect on eggs as 100 mg/ml methanolic and ethanolic extracts. In the developed eggs experiment, the highest concentrations of the three extracts were statistically the same.

**Table-1:** Ovicidal effect of *Moringa* on *Fasciola* non-embryonated eggs.

Treatment	Conc. (mg/ml)	Mean±SD				

		Non-developed eggs (%)	Hatched eggs (%)	Hatching ratio	Ovicidal activity (%)
*Moringa* methanolic extract	12.5	38.22±1.03^h^	61.78±1.03^b^	0.7±0.01^b^	31.36±1.14^h^
	25	67.68±1.49^f^	32.32±1.49^d^	0.37±0.02^d^	64.09±1.65^f^
	50	79.49±2.91^de^	20.51±2.91^ef^	0.24±0.03^e^	77.22±3.23^de^
	100	88.63±0.66^b^	11.37±0.66^h^	0.13±0.01^gh^	87.36±0.73^b^
	200	98.98±0.01^a^	1.02±0.01^i^	0.01±0^i^	98.87±0.01^a^
*Moringa* ethanolic extract	12.5	42.55±0.77^g^	57.45±0.77^c^	0.64±0.01^c^	36.17±0.85^g^
	25	43.31±1.26^g^	56.69±1.26^c^	0.63±0.14^c^	37.01±1.4g
	50	79.76±0.5d^e^	20.24±0.5e^f^	0.22±0.01^ef^	77.51±0.56^de^
	100	89.25±3.06^b^	10.75±3.06^h^	0.12±0.03^jk^	88.06±3.4^b^
	200	98.33±1.53^a^	1.67±1.53^i^	0.02±0.01^i^	98.15±1.7^a^
*Moringa* water extract	100	85.74±1.06^c^	14.26±1.06^g^	0.2±0.02^f^	84.16±1.18^c^
	200	90±2^b^	10±2^h^	0.16±0.01^g^	88.89±2.2^b^
	400	77.42±1.06^e^	22.58±1.06^e^	0.11±0.02^h^	74.91±1.18^e^
	800	91.13±0.78^b^	8.87±0.78^h^	0.1±0.01^h^	90.14±0.87^b^
Control (Dist. water)	-	10±0.91^i^	89.99±0.95^a^	1±0^a^	0±0i
Nitroxynil	100	81.84±1.57^d^	18.16±1.57^f^	0.25±0.02^e^	79.82±1.74^d^
F value		859.94	859.94	744.23	881.83
p		p<0.001	p<0.001	p<0.001	p<0.001

All data expressed as mean±SD. Means followed by different letters indicated significance. SD=Standard deviation

**Table-2:** Ovicidal effect of *Moringa* on *Fasciola* developed eggs.

Treatment	Conc. (mg/ml)	Mean±SD				
		Nondeveloped eggs (%)	Hatched eggs (%)	Hatching ratio	Ovicidal activity (%)
*Moringa* methanolic extract	12.5	46.48±1.38^h^	53.91±1.01^b^	0.61±0.02^b^	39.06±1.14^h^
	25	59.96±1.65^g^	39.73±1.43^c^	0.46±0.02^c^	55.09±1.61^g^
	50	72.02±2.11^e^	27.18±0.77^e^	0.32±0.02^e^	69.27±0.87^e^
	100	88.58±2.01^b^	11.87±1.69^h^	0.13±0.02^i^	86.59±1.91^b^
	200	94.68±2.1^a^	5.10±2.02^i^	0.06±0.02^j^	94.24±2.29^a^
*Moringa* ethanolic extract	12.5	47.06±2.67^h^	52.94±2.67^b^	0.60±0.03^b^	40.16±3.02^h^
	25	63.99±1.48^f^	36.01±1.48^d^	0.41±0.02^d^	59.3±1.67^f^
	50	80.07±0.95^d^	19.93±0.95^f^	0.23±0.01^g^	77.47±1.07^d^
	100	86.33±0.72^bc^	13.67±0.72^gh^	0.16±0.01^hi^	84.54±0.81^bc^
	200	94.10±2.35^a^	5.90±2.35^i^	0.07±0.03^j^	93.33±2.65^a^
*Moringa* water extract	100	64.4±1.68^f^	35.6±1.68^d^	0.39±0.02^d^	62.31±1.78^f^
	200	79.6±1.22^d^	20.4±1.22^f^	0.22±0.01^g^	78.4±1.29^d^
	400	84.09±2.01^c^	15.91±2.01^g^	0.17±0.02^h^	83.16±2.13^c^
	800	95.37±1.46^a^	4.63±1.46^i^	0.05±02^j^	95.09±1.54^a^
Control (Dist. water)	-	11.54±1.54^i^	88.46±1.68^a^	1±0^a^	0±0^i^
Nitroxynil	100	75.03±2.4^e^	24.97±2.4^e^	0.28±0.03^f^	71.77±2.72^e^
F value		454.07	522.79	473.97	577.01
p		p<0.001	p<0.001	p<0.001	p<0.001

All data expressed as mean±SD. Means followed by different letters indicated significance. SD=Standard deviation

Regarding the comparing the ovicidal activity of the three extracts on the eggs with independent sample t-test, significant differences were found between the 2 stages of eggs development at almost all concentrations of methanolic and aqueous extracts. Almost all concentrations of ethanolic extract had the same effect on non-embryonated and developed eggs (Table-3).

**Table-3:** Ovicidal activity of *Moringa* extracted and nitroxynil in non-embryonated and developed *Fasciola* eggs.

Treatment	Conc. (mg/ml)	Non-embryonated *Fasciola* eggs	Developed *Fasciola* eggs	T	p
*Moringa* methanolic extract	12.5	31.36±1.14	39.06±1.14	8.26	0.001**
	25	64.09±1.65	55.09±1.61	6.74	0.003**
	50	77.22±3.23	69.27±0.87	4.11	0.02*
	100	87.36±0.73	86.59±1.91	0.66	0.5^NS^
	200	98.87±0.01	94.24±2.29	3.51	0.03*
*Moringa* ethanolic extract	12.5	36.17±0.85	40.16±3.02	2.2	0.09^NS^
	25	37.01±1.4	59.3±1.67	17.73	0.000***
	50	77.51±0.56	77.47±1.07	0.05	0.96^NS^
	100	88.06±3.4	84.54±0.81	1.74	0.16^NS^
	200	98.15±1.7	93.33±2.65	2.65	0.06^NS^
*Moringa* water extract	100	84.16±1.18	62.31±1.78	17.77	0.000***
	200	88.89±2.2	78.4±1.29	7.07	0.002**
	400	74.91±1.18	83.16±2.13	5.87	0.004**
	800	90.14±0.87	95.09±1.54	4.837	0.008**
Control (Dist. water)	-	0±0	0±0	1.37	0.24^NS^
Nitroxynil	100	79.82±1.74	71.77±2.72	4.32	0.01*

All data expressed as mean±SD.

* Significant differences at p<0.05.

** Significant differences at p<0.01.

*** Significant differences at p<0.001.

^NS^Non-significant. Means followed by different letters indicated significance. SD=Standard deviation

LC_50_ of the different concentrations was calculated as shown in Table-4. LC_50_ was higher in non-embryonated eggs than developed eggs for methanolic and ethanolic extracts. Water extract exhibited a different effect as LC_50_ of aqueous extract on developed was higher than that of non-embryonated eggs.

**Table-4:** LC_50_ of different *Moringa* extracts on *Fasciola* nonembryonated and developed eggs

*Fasciola* egg	*Moringa* extract	LC_50_			

		Conc. (mg/ml)	Lower limit	Upper limit
Nonembryonated eggs	Methanol	19.98	12.42	26.42
	Ethanol	24.39	10.7	36.93
	Water	2.6	0.03	12.31
Developed eggs	Methanol	15.85	13.21	18.45
	Ethanol	12.38	4.15	16.36
	Water	60.69	44.12	76.46

## Discussion

In the current study, *M. oleifera* leaf extracts presented concentration-dependent activities against the two developmental stages of *F. gigantica* eggs tested. These results are similar to those obtained by Pessoa *et al*. [16] against *Haemonchus contortus* by the essential oil of *Ocimum gratissimum*. Furthermore, they confirmed the results obtained previously on rhabditiform larvae of *Ancylostoma caninum* by Poné *et al*. [17] and on eggs of the nematode *A. caninum* with the extracts of the shrub *Canthium mannii* by Poné *et al*. [18].

Alcoholic and aqueous extracts of *M. oleifera* represented high anthelmintic activity on eggs. In addition, all extracts rendered most of non-embryonated *F. gigantica* eggs undeveloped suggesting that the bioactive compounds were lethal to the blastomeres. Furthermore, Rastogi *et al*. [8] found that the ethanolic extract of *M. oleifera* showed strong anthelmintic activity at 25, 50, and 100 mg/ml against Indian earthworm. On the contrary, *M. oleifera* did not give satisfactory *in vitro* anthelmintic results on *Ascaris suum* with Peter and Deogracious [19] with a median effective dose more than 50 mg/ml in the initial screening.

The ovicidal activities observed in the present study using different extracts might be attributed to the presence of saponins, steroids, carbohydrates, alkaloids, tannins, and flavonoids which were reported in *M. oleifera* leaves [20,21]. Previously, it was suggested that tannins and saponins possibly penetrated the egg affecting the morula [22] and stop hatching [23] of *H. contortus* eggs.

In the current study, there were no statistically significant differences between ovicidal activity of *M. oleifera* methanolic and ethanolic extracts in non-embryonated and developed eggs. The probable reason for the minor differences between aqueous and alcoholic extracts could be due to variation in solubility of the active compounds in the solvent. Infused aqueous extract and ethanolic extract of *M. oleifera* tested by Tayo *et al*. [24] on *H. contortus, in vitro*, presented comparable activity on eggs. The observed similar activity of ethanolic extract and infused aqueous extract of *M. oleifera* leaves on eggs could be due to the presence of similar or related chemicals having ovicidal properties in approximately equivalent proportions. On the contrary, Poné *et al*. [18] found that the ethanol extract was more potent on the eggs when compared to hot and cold water extracts.

Based on LC_50_ values, water extract presented the highest ovicidal activity since it registered the lowest values of 2.6 mg/ml on immature non-embryonated eggs suggesting that non-embryonated eggs were more susceptible to aqueous extract than developed eggs. On the other hand, developing eggs were more susceptible to ethanolic extract than non-embryonated eggs as ethanolic extract had the lowest LC_50_ (12.38 mg/ml). These results agreed with Tayo *et al*. [24] who found that ethanolic extracts of *M. oleifera* leave presented the highest activity on late stages of development of *H. contortus* eggs since it registered the lowest values of LC_50_. They disagreed with Poné *et al*. [18] found that LC_50_ value of the ethanol extract of *C. mannii* was relatively low, indicating that this extract is more active on non-embryonated eggs than aqueous extracts. This conflict might be due to they used *H. contortus* eggs, and in the current study, *F. gigantica* eggs were used. Although not quite exact on how *M. oleifera* inhibit egg embryonation, Sreelatha *et al*. [25] explained that *M. oleifera* leaf extract could induce cellular apoptosis, morphological change and DNA fragmentation in a type of human cancer cell.

## Conclusion

The current study introduced *M. oleifera* leaf aqueous extract which offered a promising ovicidal effect on *F. gigantica* eggs. The future scope involves the need of *in vivo* study to enlighten the therapeutic potential of *M. oleifera* extracts in treating *F. gigantica* infection. Furthermore, isolation of phytoconstituents responsible for these activities is required.

## Authors’ Contributions

This study was designed and supervised by AGH. While, SEH, EEE and DA carried out extracts preparation. AMM and NIT collected the samples. KNA, SEH, EEE and DA examined gallbladders. KNA, SEH, DA and EEE carried out the application of plant extracts on eggs. DA analyzed and interpreted the data. All authors have prepared, read and approved the final manuscript.
